# Тиротоксический криз у пациентки с сочетанием болезни Грейвса и сахарного диабета 1 типа

**DOI:** 10.14341/probl13688

**Published:** 2026-09-08

**Authors:** Л. И. Данилова, Г. Г. Короленко, М. Л. Лущик, О. В. Чаплинская, И. И. Бурко

**Affiliations:** Белорусский государственный медицинский университет Беларусь; Belarusian State Medical University Belarus; Гомельская городская клиническая больница №3 Беларусь; Gomel City Clinical Hospital №3 Belarus

**Keywords:** болезнь Грейвса, диабетический кетоацидоз, сахарный диабет 1 типа, тиреотоксикоз, тиреотоксический криз, Graves’ disease, diabetic ketoacidosis, type 1 diabetes mellitus, thyrotoxicosis, thyrotoxic crisis, thyroid gland

## Abstract

Тиротоксический криз (ТК) представляет собой редкое, но потенциально жизнеугрожающее осложнение декомпенсированного тиротоксикоза, требующее немедленного вмешательства. Диагностика ТК основывается на выраженной клинической картине тяжелого гипертиреоза в сочетании с провоцирующими факторами. Терапию следует начинать при первых подозрениях на развитие криза, без ожидания результатов гормонального анализа, получение которых может занять значительное время. В статье представлен клинический случай пациентки с сочетанием болезни Грейвса и сахарного диабета 1 типа, у которой ТК был своевременно распознан и эффективно купирован. Провоцирующим фактором выступил диабетический кетоацидоз. Описанный случай подчеркивает важность ранней диагностики и интенсивной терапии для улучшения прогноза при ТК.

## Abstract (en)

Thyrotoxic crisis (TC) is a rare but potentially life-threatening complication of decompensated thyrotoxicosis that requires immediate medical intervention. The diagnosis of TC is based on a clinical picture of severe hyperthyroidism in combination with precipitating factors. Treatment should be initiated at the first suspicion of crisis development, without waiting for the results of hormonal analysis, which may take considerable time to obtain. This article presents a clinical case of a female patient with coexisting Graves’ disease and type 1 diabetes mellitus, in whom TC was promptly recognized and effectively managed. The precipitating factor was diabetic ketoacidosis. This case highlights the importance of early diagnosis and intensive therapy to improve the prognosis of patients with TC.

## АКТУАЛЬНОСТЬ

Тиреотоксический криз (ТК) — это острое неотложное состояние, относящееся к числу критических ситуаций в клинической эндокринологии. Это редкое, но угрожающее жизни осложнение, характеризующееся внезапным усилением симптомов тиреотоксикоза и развитием признаков полиорганной недостаточности [1].

ТК по-прежнему представляет собой серьезную диагностическую и терапевтическую проблему. Согласно статистическим данным, полученным в США и Японии, уровень смертности при ТК превышает 10%, особенно в случаях его несвоевременного распознавания и отсутствия активного лечения [2][3]. Высокая летальность при ТК обусловлена развитием сердечной недостаточности, фатальных аритмий, недостаточностью коры надпочечников, тромбоэмболическими осложнениями, тяжелым судорожным синдромом с отеком головного мозга, печеночной недостаточностью [4].

Авторы большинства публикаций обращают внимание на тот факт, что ТК не имеет специфических лабораторных маркеров, что затрудняет диагностику. Существующие диагностические шкалы основаны преимущественно на клинических признаках и степени выраженности симптомов [3][5]. В зависимости от степени системной декомпенсации выделяют 2 стадии: раннюю — надвигающийся «шторм» и фактический кризис — «тиреоидный шторм» [5].

Точные механизмы, лежащие в основе развития ТК, изучены недостаточно. Основным пусковым моментом развития ТК считают острое и значительное повышение концентрации свободных фракций тиреоидных гормонов, что приводит к избыточной активации симпатоадреналовой системы и развитию острой надпочечниковой недостаточности [6]. Относительная острая надпочечниковая недостаточность развивается в результате ускоренного клиренса глюкокортикоидов вследствие чрезмерной активации метаболических процессов. Избыточная концентрация катехоламинов сопровождается повышением чувствительности периферических тканей к их действию. При этом отсутствует строгая корреляция между уровнем тиреоидных гормонов в крови и тяжестью тиреотоксического криза [5].

Клиническая картина при ТК схожа с проявлениями неосложненного тиреотоксикоза, однако отличается большей выраженностью симптомов и стремительным прогрессированием, часто сопровождающимся развитием полиорганной недостаточности. Клинические проявления ТК могут значительно усиливаться по сравнению с типичным течением тиреотоксикоза. Непереносимость высоких температур при ТК может смениться гипертермией, достигающей 40–41 °C. Синусовая тахикардия приобретает чрезмерный характер, вплоть до развития фибрилляции предсердий, и может сопровождаться сердечной недостаточностью. Диарея, наблюдаемая при тиреотоксикозе, может переходить в профузный понос, нередко наблюдается рвота. Эмоциональная неустойчивость может смениться психозом или комой [7].

Согласно литературным данным, ТК может быть спровоцирован рядом факторов, вызывающих острый стрессовый ответ организма. К числу таких триггеров относят: эмоциональный стресс; травмы; хирургические вмешательства по поводу сопутствующих заболеваний; инфекционные процессы; диабетический кетоацидоз и гипогликемию; внезапное необоснованное прекращение тиреостатической терапии; массивную нагрузку йодом (например, внутривенное введение контрастных веществ при рентгенологических исследованиях); терапию радиоактивным йодом; грубую пальпацию щитовидной железы (ЩЖ); тромбоэмболию легочной артерии; острое нарушение мозгового кровообращения (ОНМК); инфаркт миокарда; сердечную недостаточность; роды и другие состояния, сопровождающиеся выраженным физиологическим стрессом [8][9].

Диагностика ТК основывается на наличии клинических признаков тяжелого некомпенсированного тиреотоксикоза. При этом важным диагностическим критерием является наличие провоцирующих факторов.

Терапия ТК должна начинаться немедленно при подозрении на ТК, без ожидания результатов гормонального анализа, получение которых может занять значительное время Раннее выявление, точнее — своевременное предположение о возможном развитии ТК, быстрая диагностика и интенсивное лечение существенно повышают шансы на благоприятный исход. В то же время отсутствие специфических биологических маркеров и схожесть симптомов с проявлениями провоцирующего заболевания затрудняют диагностику ТК. В качестве иллюстрации своевременной диагностики и лечения ТК представлен клинический случай пациентки с сочетанием болезни Грейвса (БГ) и сахарного диабета 1 типа (СД 1).

## ОПИСАНИЕ СЛУЧАЯ

Пациентка Т., 29 лет, 18 декабря 2024 г. в экстренном порядке была доставлена в приемное отделение больницы по месту жительства с диагнозом: «Сахарный диабет 1 типа, гипергликемия».

Три года назад у нее была диагностирована БГ. В течение полутора лет она наблюдалась по поводу СД1. Со слов матери, пациентка вводила инсулин, принимала тирозол, а накануне употребляла алкоголь. Спустя пару дней после эпизода злоупотребления алкоголем у нее появилась тошнота, многократная рвота. Пациентка стала вести себя неадекватно и возбужденно.

В ходе объективного осмотра состояние женщины было оценено как тяжелое, она была недоступна для контакта. Кожные покровы гиперемированы, горячие на ощупь, влажные, выражен акроцианоз с мраморным рисунком на конечностях. Температура тела — 39,5 °C. Слизистые оболочки рта и языка были сухие, язык покрыт коричневым налетом. Отмечался выраженный запах ацетона в выдыхаемом воздухе пациентки. Аускультативно над легкими выслушивалось везикулярное дыхание, ЧД — 24/мин. Тоны сердца были ритмичные, приглушены. АД — 130/90 мм рт. ст. на обеих руках, ЧСС — 150/мин. Периферические отеки отсутствовали. При пальпации ЩЖ была увеличена, эластичная, однородная, безболезненная.

Бригадой скорой медицинской помощи предпринята попытка медикаментозной седации, однако ожидаемого результата достигнуто не было.

В связи с выраженной тяжестью состояния пациентку госпитализировали в отделение интенсивной терапии. Ввиду неэффективности дыхания была начата искусственная вентиляция легких (ИВЛ), медикаментозная седация продолжена.

По данным лабораторного исследования выявлена гипергликемия (глюкоза венозной крови — 34 ммоль/л) и выраженные изменения кислотно-основного состояния (КОС) — рН 6,8; содержание лактата — 4,5 ммоль/л; выраженный дефицит буферных оснований (Base Excess — ВЕ) — -31,1 ммоль/л.

Отмечены высокие уровни прокальцитонина — 0,55 нг/мл (норма до 0,05), лейкоцитоз — 16,6*10⁹/л (4,0–9,0), концентрация С-реактивного белка (СРБ) — 8,9 мг/л (норма до 6,0), а также повышение концентрации креатинина крови — 134 мкмоль/л. Остальные лабораторные показатели находились в пределах референсных значений.

Гормональный анализ выявил значительные нарушения тиреоидного статуса: тиреотропный гормон (ТТГ) <0,001 мМЕ/л (0,35–4,94), свободный тироксин (свТ4) >64,35 пмоль/л (9,0–9,0), антитела к тиреопероксидазе (АТ ТПО) >1000 (0,35–4,94 МЕ/мл), антитела к рецептору ТТГ (АТ рТТГ) — 40,7 МЕ/л (0–2,65).

Учитывая выраженную гипертермию и воспалительный характер изменений в анализах крови, проведены инструментальные методы обследования. Компьютерная томография органов грудной и брюшной полостей и рентгенография легких данных за воспалительный процесс не выявили.

Заключение УЗИ щитовидной железы: расположение обычное; контуры железы неровные, четкие; эхогенность неравномерно снижена; эхоструктура неоднородна за счет фиброзной тяжистости; выражена васкуляризация. Суммарный объем железы составил 67,79 см³.

Тяжесть состояния пациентки определялась сочетанием тиреотоксикоза, выраженного диабетического кетоацидоза (ДКА) и алкогольной интоксикации.

Пациентке проводилась инфузионная терапия, интенсивная инсулинотерапия с ежечасным мониторингом гликемии и коррекцией дозы инсулина по результатам контроля, а также жаропонижающая, антикоагулянтная и антибактериальная терапия. В первые сутки через назогастральный зонд введено 40 мг тирозола.

На фоне проводимой терапии через 24 часа ДКА купирован: показатели КОС нормализовались, гликемия стабилизировалась на уровне 8–11 ммоль/л. Однако сохранялась выраженная гипертермия (39–40 °C) и чрезмерная тахикардия при отсутствии электролитных нарушений (ЧСС — 150–170/мин). На фоне продолжающейся ИВЛ и медикаментозной седации у пациентки возник эпизод фибрилляции предсердий, купированный внутривенным введением метонорма (5 мл). При неврологическом осмотре клинических данных за острую неврологическую патологию не выявлено. По результатам лабораторных исследований отмечена тенденция к снижению лейкоцитоза; уровни прокальцитонина и СРБ не превышали верхний предел референсного интервала.

С учетом выраженной клинической симптоматики тиреотоксикоза и результатов гормонального исследования крови был проведен расчет по шкале BWPS (Burch–Wartofsky), по результатам которого набрано 80 баллов, что свидетельствовало о развитии у пациентки ТК.

Был выставлен клинический диагноз: «Аутоиммунный полигландулярный синдром 3 типа: Болезнь Грейвса с диффузным зобом и объемом ЩЖ 67,79 см³, тиреотоксикоз тяжелой степени. Тиреотоксический криз. Тиреотоксическая энцефалопатия. Тиреотоксическая миокардиопатия с пароксизмом фибрилляции предсердий. Н1. Сахарный диабет 1 типа (HbA1c 8,8% от 19.12.2025 г). Диабетический кетоацидоз тяжелой степени».

В отделении интенсивной терапии проводилось следующее лечение ТК: тирозол — 80 мг внутрь через назогастральный зонд однократно, затем 30 мг каждые 8 часов; пропранолол — 40 мг каждые 4 часа, раствор Люголя — по 5 капель внутрь каждые 8 часов через назогастральный зонд (через 1 час после введения тиреостатика); гидрокортизон — 300 мг внутривенно одномоментно, далее по 100 мг каждые 8 часов. Продолжена инфузионная терапия и инсулинотерапия.

На фоне медикаментозной терапии к 4-м суткам пребывания в отделении интенсивной терапии температура тела снизилась до 37,5 °C. Гормональные исследования показали выраженную тенденцию к снижению уровня свТ4. В связи с улучшением состояния пациентки была уменьшена доза тирозола до 40 мг в сутки, скорректирована доза пропранолола, парентеральное введение метилпреднизолона заменено на прием таблетированной формы перорально (64 мг через день). Продолжена терапия раствором Люголя.

На 12-й день комбинированной терапии тиреостатиками, препаратом йода и метилпреднизолоном у пациентки был достигнут лабораторный эутиреоз (свТ4 — 15,1 пмоль/л). В связи с этим принято решение отменить раствор Люголя и продолжить терапию тирозолом в дозе 30 мг/сут под контролем свободных фракций тиреоидных гормонов.

31.12.2024 г. проведена трахеостомия. ИВЛ продолжена в связи с развитием двусторонней полисегментарной пневмонии.

22.01.2025 г. для дальнейшего лечения пациентка переведена в эндокринологическое отделение. Спустя месяц выполнена тотальная тиреоидэктомия. С заместительной целью был назначен левотироксин натрия.

## ОБСУЖДЕНИЕ

В рассматриваемом клиническом случае у пациентки наблюдалось сочетание нескольких взаимно отягощающих критических состояний: ДКА тяжелой степени на фоне СД1 и алкогольной интоксикации, а также ТК, развившийся вследствие несоблюдения режима приема тиреостатиков по поводу БГ.

Наличие двух аутоиммунных эндокринных заболеваний позволило сформулировать диагноз аутоиммунного полигландулярного синдрома (АПС) 3 типа. Следует отметить, что манифестация сахарного диабета произошла через 1,5 года после дебюта тиреотоксикоза, что подтверждает целесообразность раннего выявления островковых аутоантител у молодых лиц с иммунным тиреотоксикозом [10].

Провоцирующим фактором развития ДКА явилось употребление алкоголя при отсутствии адекватной заместительной инсулинотерапии СД1, что, в свою очередь, привело к декомпенсации тиреотоксикоза и клиническому проявлению ТК. Не исключается несоблюдение пациенткой режима приема тирозола.

Основанием для подозрения на развитие данного тяжелого и редкого осложнения стало отсутствие клинического улучшения после купирования клинико-лабораторных проявлений ДКА, несмотря на нормализацию КОС, волемического статуса и уровня гликемии. Пациентка оставалась в медикаментозной седации; сохранялась гипертермия без лабораторных и инструментальных признаков воспалительного процесса.

Повышенные в первый день значения СРБ и прокальцитонина нормализовались в процессе купирования ДКА. По современным данным, состояние ДКА сопровождается повышением уровня провоспалительных цитокинов, СРБ и биомаркеров перекисного окисления липидов независимо от наличия явной инфекции или сердечно-сосудистой патологии [10][11].

Важно подчеркнуть, что по данным опубликованных клинических наблюдений, для постановки диагноза ТК не требуется наличие крайне высоких значений свободных фракций тиреоидных гормонов. Диагноз устанавливается преимущественно на основании клинической картины и наличия провоцирующих факторов [8].

В 1993 г. H.B. Burch и L. Wartofsky разработали и предложили балльную шкалу (Burch–Wartofsky Point Scale, BWPS), предназначенную для диагностики ТК. Она основана на количественной оценке клинических проявлений со стороны сердечно-сосудистой, пищеварительной и центральной нервной систем (табл. 1).

**Table:** Таблица 1. Балльная шкала диагностики ТК по Бурху–Вартофскому [5] Примечание. <25 баллов — ТК маловероятен; 25–44 балла — высокая вероятность развития ТК; >44 баллов — ТК.

Нарушение терморегуляции	Сердечно-сосудистые проявления
Температура тела, °C	Баллы	Тахикардия, уд/мин	Баллы
37,2–37,7	5	99–109	5
37,8–38,2	10	110–119	10
38,3–38,8	15	120–129	15
38,9–39,4	20	130–139	20
39,5–39,9	25	>140	25
>40,0	30	Фибрилляция предсердий	10
Сердечная недостаточность	Неврологические проявления
Степень тяжести	Баллы	Степень тяжести	Баллы
Легкая		Легкая	
-отеки ног	5	- возбуждение	10
Умеренная		Умеренная	
-хрипы в нижних отделах легких	10	- делирий, психоз, ступор	20
Тяжелая		Тяжелая	
- отек легких	15	- судороги, кома	30
Гастроинтестинальные, печеночные проявления	Провоцирующий фактор
Степень тяжести	Баллы		Баллы
Умеренная			
- диарея, тошнота, рвота, боль в животе	10	“+”	10
Тяжелая			
- желтуха	20	“–”	0

Шкала BWPS представляет собой эмпирически полученную систему оценки, которая учитывает тяжесть симптомов полиорганной декомпенсации, включая терморегуляторную дисфункцию, тахикардию/фибрилляцию предсердий, нарушения сознания, застойную сердечную недостаточность и гастрогепатическую дисфункцию, а также роль провоцирующих факторов [5]. При сумме баллов по шкале Burch–Wartofsky результат >44 свидетельствует о наличии ТК и требует безотлагательных лечебных мероприятий. Показатели от 25 до 44 баллов расцениваются как высокая вероятность развития ТК. Результат <25 баллов соответствует маловероятному развитию криза.

В 2012 г. Японская тиреоидологическая ассоциация (JTA) предложила обновленные диагностические критерии ТК, в которых особое значение придается нарушению сознания как ключевому диагностическому признаку, превосходящему по значимости другие симптомы [7]. В представленном случае именно чрезмерное возбуждение, неадекватное состояние и температурная реакция пациентки стали доминирующими в клинической картине, что позволило заподозрить развитие ТК.

Таким образом, клиническая настороженность и тщательная оценка симптомов тиреотоксикоза, возникающих на фоне провоцирующих факторов, играют решающую роль в своевременной диагностике ТК. В случаях, когда невозможно достоверно установить, связаны ли симптомы с сопутствующей патологией или с самим кризом, их следует расценивать как проявления ТК.

Как только стало очевидным, что пациентка находится в состоянии ТК, был незамедлительно пересмотрен протокол лечения. Согласно существующим Клиническим рекомендациям [1][7][9][12] при лечении пациентов с ТК следует использовать комплексный подход, включающий патогенетическую терапию тиреостатиками в высоких дозах, препараты неорганического йода, β-адреноблокаторы. Для коррекции нейрогормонального статуса организма в условиях стресса и относительной надпочечниковой недостаточности необходимо вводить высокие дозы глюкокортикоидов. Наряду с этим проводится терапия, направленная на снижение гипертермии, купирование судорожного синдрома и поддержание жизненно важных функций.

К препаратам первой линии лечения ТК относятся препараты из группы тиреостатиков: метимазол (тиамазол), карбимазол, пропилтиоурацил, механизм действия которых основан на ингибировании органификации йода и конденсации йодтиреозильных остатков путем воздействия на тиреоидную пероксидазу. Это приводит к прекращению синтеза новых тиреоидных гормонов. Блокада органификации йода достигается уже в течение первого часа после назначения любого из доступных тиреостатиков [8]. Рекомендуемые дозы тиамазола при ТК составляют 60–80 мг однократно в начальной дозе, с последующим введением по 30 мг каждые 6–8 часов до достижения клинической стабилизации. При отсутствии сознания препарат вводится в измельченном виде через назогастральный зонд. Следует учитывать, что на сегодняшний день не существует доступных форм тиреостатиков для парентерального применения.

Эффект подавления секреции тиреоидных гормонов тиреостатиками проявляется через 24 часа, блокада синтеза — через несколько дней [13].

К препаратам второй линии, усиливающим тиреостатический эффект тионамидов, относятся йодиды. Механизм их действия обусловлен блокадой синтеза тиреоидных гормонов за счет нарушения процессов органификации йода — эффект Вольффа-Чайкоффа. Йод поступает в цитоплазму фолликулярных клеток ЩЖ благодаря действию натрий-йодного переносчика белковой природы (натрий-йод симпортера — NIS). Высокие дозы йода блокируют его функцию, а также функцию ТПО. Считается, что транзиторный характер эффекта Вольффа-Чайкоффа обусловлен снижением экспрессии мРНК, кодирующей NIS [14]. Тиреостатический эффект йодида при ежедневном приеме в среднем захватывает 10–14 дней, далее наблюдается «ускользание» [15][16].

В клинической практике используют концентрированный раствор йодида калия (5 капель, что соответствует 0,25 мл или 250 мг) внутрь каждые 6–8 часов или, как альтернативный препарат, раствор Люголя в аналогичной дозировке и интервале [1]. Препараты йода рекомендуется назначать не ранее чем через час после приема тиреостатиков и достижения снижения, а в ряде случаев — блокады синтеза тиреоидных гормонов. Считается, что в противном случае это может привести к увеличению их синтеза в связи с временной активацией йодозависимых механизмов и ухудшению течения тиреотоксикоза [8][17]. По данным ряда авторов, рекомендуемая суточная доза йода составляет до 200 мг, при этом более высокие дозы назначаются пациентам с подозрением на снижение желудочно-кишечной абсорбции [9][17]. В литературе описаны также ректальный и сублингвальный способы введения препаратов йода [18].

Содержание тиреоидных гормонов в крови при использовании неорганического йода снижается быстро, обычно в течение 7–10 дней, что подтверждается и нашим клиническим наблюдением (рис. 1).

**Figure:**
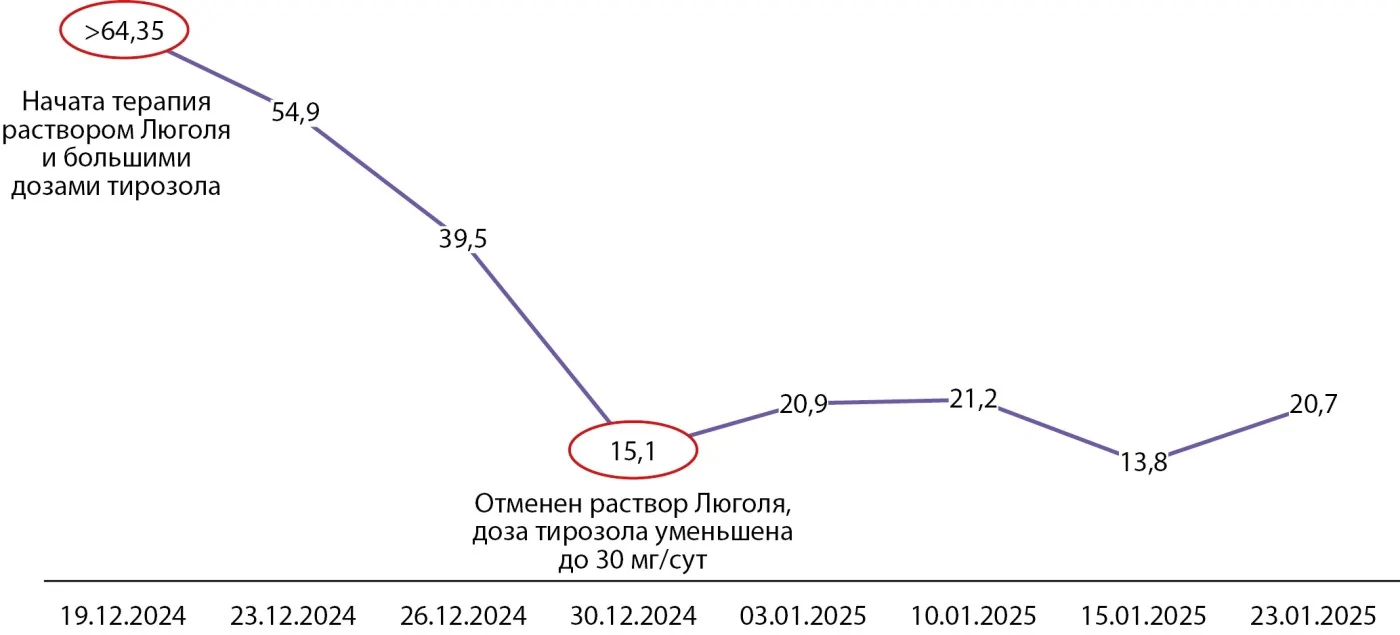
Рисунок 1. Динамика уровня свT4 (9,0–19,0 пмоль/л) в сыворотке крови пациентки Т., 29 лет, на фоне проводимой медикаментозной терапии.

Однако после достижения терапевтического эффекта, несмотря на продолжение приема йодидов, может развиваться эффект «ускользания», обусловленный восстановлением чувствительности натрий-йодного симпортера и «прорывом» синтеза тиреоидных гормонов в избыточном количестве. В этом случае возможно развитие синдрома Йод-Базедова [19]. С другой стороны, в ряде исследований было показано, что совместное использование препаратов йода и тиреостатиков позволяет значительно снизить дозу последних при тяжелом тиреотоксикозе для достижения эутиреоза, что уменьшает риск токсических эффектов и не приводит к обострению тиреотоксикоза [16][20][21].

В клинической практике после 10–14 дней терапии препаратами йода при БГ обычно показано хирургическое лечение. Однако в представленном случае тяжелое состояние пациентки не позволило выполнить хирургическое вмешательство в указанные сроки. У пациентки развилась двусторонняя пневмония, потребовавшая респираторной поддержки с помощью ИВЛ. На протяжении всего периода наблюдения осуществлялся регулярный мониторинг свободных фракций тиреоидных гормонов для своевременной коррекции терапии. Увеличения значений этих показателей не отмечалось до выполнения тотальной тиреоидэктомии.

В терапии ТК используют β-адреноблокаторы. Препаратом выбора является пропранолол в дозе 40 мг каждые 4 часа. Он обладает селективной способностью ингибировать дейодиназу 1 типа, что приводит к снижению конверсии Т4 в Т3, а также снижает чувствительность симпатической нервной системы к катехоламинам при высоких концентрациях тиреоидных гормонов. В результате уменьшается выраженность таких симптомов, как тремор, сердцебиение, беспокойство [1][12].

Одной из ключевых патогенетических стратегий в лечении ТК остается применение ГК в высоких дозах. Их назначение направлено, прежде всего, на коррекцию относительной надпочечниковой недостаточности и патофизиологического феномена острого стресса, а также на снижение конверсии Т4 в Т3 [1][9]. Наиболее часто применяют гидрокортизон — 300 мг внутривенно болюсно с последующим введением по 100 мг каждые 8 часов. В качестве альтернативы может использоваться метилпреднизолон (250–500 мг/сут внутривенно) с постепенным снижением дозы через 2–3 суток в зависимости от динамики клинической симптоматики. Альтернативой также является дексаметазон в дозе 8 мг/сут внутривенно [1][9].

Одновременно с патогенетической терапией ТК проводится коррекция гипертермии, водно-электролитных нарушений, психомоторного возбуждения (вплоть до острого психотического состояния), судорожного синдрома, сердечно-сосудистых нарушений. При отсутствии эффекта от осуществляемой терапии целесообразно проведение плазмафереза или экстренного хирургического вмешательства (тотальная тиреоидэктомия) [22].

## ЗАКЛЮЧЕНИЕ

В представленном клиническом случае АПС 3 типа наблюдалось сочетание двух жизнеугрожающих состояний — ДКА и тяжелого тиреотоксикоза с развитием ТК. Клинические проявления ДКА маскировали дебют ТК, что затрудняло своевременную диагностику. Тем не менее комплексная оценка клинической картины позволила заподозрить развитие ТК и начать его своевременную терапию. Ухудшение течения тиреотоксикоза у пациентов с БГ или другими причинами тиреотоксикоза на фоне интеркуррентного заболевания или действия любого другого провоцирующего фактора должно настораживать в отношении возможного развития ТК. При наличии симптомов, соответствующих критериям шкалы Burch-Wartofsky, и при подсчете баллов, указывающих на высокий риск или наличие ТК, необходимо немедленно начинать интенсивную терапию этого критического состояния.

## ДОПОЛНИТЕЛЬНАЯ ИНФОРМАЦИЯ

Источники финансирования. Работа выполнена по инициативе авторов без привлечения финансирования.

Конфликт интересов. Авторы декларируют отсутствие явных и потенциальных конфликтов интересов, связанных с содержанием настоящей статьи.

Участие авторов. Все авторы одобрили финальную версию статьи перед публикацией, выразили согласие нести ответственность за все аспекты работы, подразумевающую надлежащее изучение и решение вопросов, связанных с точностью или добросовестностью любой части работы.

Согласие пациента. Пациентка добровольно подписала информированное согласие на публикацию персональной медицинской информации в обезличенной форме в любых медицинских журналах, включая «Проблемы эндокринологии».
